# Pathology of Asthma

**Published:** 1852-07

**Authors:** 


*Pathology af Asthma.*—Amongst the disturbances that may give rise to asthma, Wunderlich mentions:

1. Those occurring in the respiratory organs. Thus, congestion, extensive tubercular or cancerous deposits, emphysema, œdema, chronic bronchial catarrh, affections of the glottis, pleural adhesions, &c., have been mistaken by some writers for asthma itself, and have been regarded by others as causes of that affection. These are often complications of asthma, but we agree with Wunderlich in doubting whether they ever actually give rise to it.

2. Asthmatic attacks sometimes appear to depend on changes in the brain or spinal cord and their membranes, and on tumors pressing on the pneumogastric nerve. Alteration of structure in the brain near the origin of that nerve, chronic myelitis, ossification of the pulmonary plexus, and a tuberculous condition of the bronchial glands pressing on the pneumogastric nerve, have been described by different writers as causes of asthma. Wunderlich narrates an obstinate case of asthma recurring every week or two for several years. The patient was a man who had a small tumor under the anterior origin of the sterno-cleido-mastoid muscle, which gradually extended beneath the clavicle, and probably pressed upon the pneumogastric nerve. There was also slight emphysema, but not enough to account for the severe asthmatic attacks, which usually lasted for from three to eight days, and occurred at very short periods.

3. Affections of the heart and pericardium, and aneurism of the large thoracic vessels, have often been described as causes of asthma. Rostan even went so far as to maintain that asthma is always a symptom of an organic disease of the heart or of the large vessels. We should think that these diseases would primarily induce mechanical dyspnœa, rather than spasmodic asthma; they may, however, in a secondary manner, irritate the pneumogastric nerve, and thus actually give rise to the disease in question.

4. It has been generally assumed that deranged conditions of the stomach and intestinal canal, the liver, and the genito-urinary organs, may directly induce asthma. Whether this be the case or not, it is at all events certain that errors of diet, sexual excesses, and derangements of the digestive system, increase and aggravate the attacks of persons liable to asthma.

5. Certain conditions of the blood may cause asthmatic attacks. A superabundance of blood, or blood too rich in solid constituents, may give rise to symptoms that may be mistaken for asthma, although they are in reality symptoms pertaining to pulmonary congestion; and, on the other hand, a deficient supply of blood, or blood that is very poor in solid constituents, may give rise to symptoms that may be mistaken for asthma, although they are in reality symptoms pertaining to pulmonary congestion; and, on the other hand, a deficient supply of blood, or blood that is very poor in solid constituents, may give rise to symptoms which are, however, rather those of spasm of the glottis and of the diaphragm, than those of true asthma. The paroxysms of dyspnœa occurring in putrid and purulent poisoning of the blood, do not constitute true asthma. Wunderlich seems to doubt whether, the attacks of dyspnœa, which are so common when the kidneys are inert and the urinary constituents are retained in the blood, are really deserving of being considered asthmatic; indeed, he plainly expresses it as his opinion that in all probability the *asthma urinosum* of the older physicians and of Schonlein, is nothing more than pulmonary emphysema. As, however, he grants that lead, when introduced into the system, often produces asthma, and that other poisons not unfrequently do so, we see no reason why he should object to allow that the dyspnœa produced by the accumulation of urea in the blood is of an asthmatic nature.

Whatever may be the anatomical changes which are found in examining the bodies of persons who have suffered from asthma, the disease seems essentially to consist in a temporary and paroxysmal spasmodic constriction of the smallest bronchial canals (for the cartilaginous structure of the larger tubes will not allow of their being thus affected.)

Asthma is seldom the immediate cause of death; but frequent attacks give rise to hypertrophy of the bronchial tubes, emphysema of the lungs, or certain forms of heart disease, which modify and aggravate the original affection, and hasten a fatal termination.

We find very few points of novelty in any of our authors in relation to treatment. Wunderlich recommends that during the paroxysm, if it comes on suddenly and with great intensity, the bowels should be emptied by a powerful injection; and he seems to think that there are many cases in which venesection would be serviceable. As, however, he adds, the arguments for and against bleeding are often nearly balanced, it is sometimes expedient to open a vein, and either to close it almost directly, or to allow the blood to flow, according to the immediate effect produced on the patient. The general testimony is, however, strongly against venesection. If the patient can drink, he should take a little warm tea containing opium, stramonium, or lobelia inflata. Strong black coffee and iced drinks have also been recommended. In those cases where the attack is more gradual, an emetic often gives great relief, and sometimes at once cuts short the paroxysm. Ipecacuanha in small doses is sometimes useful; but what Wunderlich has found most useful is a combination of tartar emetic with large doses of opium (from half a grain to one grain every hour). Stramonium smoked with an equal quantity of tobacco relieves the pains which are usually felt in the chest, but is of no other use. (This, at least, is Wunderlich's experience, who adds, that the much-praised camphor cigarettes are even less efficacious.) Constatt, as we have already had occasion to mention, enters more fully into the consideration of the individual therapeutic agents, than Wunderlich or any of our authors. We give his observations on a few of the medicines that have at different times had a high reputation in asthma:

"The Datura Stramonium has obtained a character as a specific against asthma (Krimer, Cruveilheir, Reimer, Zeigler, Ward, Marcet, Meyer, Helm, Cunningham, Hegewisch, Ferrus, Elliotson.) It is given in powder, in gradually increased doses, from two to twelve grains; in extract, from a quarter of a grain to four grains; or in tincture, from five to twenty drops. We frequently recommend the stalks and leaves to be smoked, the patient being directed to swallow his saliva. Hegewisch orders that the number of pipes should be increased, till all the urgent thoracic symptoms have disappeared; if a relapse comes on, the patient must again begin with one or two pipes, and proceed as before. Unfortunately, asthmatic patients are often unable to inhale the smoke. The Datura fastuosa has been employed by Adams in the form of a tincture, of which thirty or forty drops were to be taken every two hours; and by Skipton in the form of decoction of its bark.

"The Lobelia inflata [Indian tobacco] was first employed by American physicians, and has recently obtained a high reputation as a remedy for asthma [Eberle, Barton, Chapman, Whitlaw, Andrews, Bidault, De Villiers, Reece, Sigmond, Elliotson, Newmann, Forbes, Morelli, Noack]. We usually give twenty or thirty drops of the tincture of the leaves at the commencement of the paroxysm, or shortly before an attack is expected; more rarely we give the powder in doses of fifteen or twenty grains. It appears to exert a specific influence on the respiratory nervous system.

"Common tobacco acts in a similar manner; it may be smoked or given in the form of tincture. . . . . . . . The Bignonia catalpa (indigenous in Carolina) was described as a specific by Kampfer and Taubery; and has been more recently extolled by Brera, Brudent, Antomarchi, and Antonisca." (Constatt's Handbuch der medicinischen Klinik,' vol. iii. part 2, p. 450.)—*Ibid.*

